# Differences in self-monitored, blood glucose test strip utilization by therapy for type 2 diabetes mellitus

**DOI:** 10.1007/s00592-015-0823-z

**Published:** 2016-03-14

**Authors:** Ruben Tavares, Marc Duclos, Marie-Josée Brabant, Daniella Checchin, Nevzeta Bosnic, Katherine Turvey, Jorge Alfonso Ross Terres

**Affiliations:** GlaxoSmithKline, 7333 Mississauga Road North, Mississauga, ON L5N 6L4 Canada; IMS Brogan, a unit of IMS Health, Kirkland, QC Canada; IMS Brogan, a unit of IMS Health, Ottawa, ON Canada; IMS Brogan, a unit of IMS Health, Mississauga, ON Canada; GlaxoSmithKline, King of Prussia, Philadelphia, PA USA

**Keywords:** Blood glucose self-monitoring, Type 2 diabetes mellitus, Utilization

## Abstract

**Aims:**

To determine whether blood glucose test strip (BGTS) utilization in patients with type 2 diabetes (T2D) is associated with the type of diabetes therapy, classified according to hypoglycemic risk.

**Methods:**

A retrospective, longitudinal (2006–2012) study of Canadian private drug plans (PDP) and Ontario Public Drug Programs (OPDP) prescription claims was conducted. Analyses were restricted to patients with T2D with or without a claim for BGTS. Daily BGTS utilization (TS/patient/day) was evaluated by diabetes therapy classified by hypoglycemic risk. Multivariate analyses were conducted to identify determinants of BGTS utilization.

**Results:**

The T2D cohort comprised 5,759,591 observations from 1,949,129 claimants. Mean BGTS utilization was 0.84 TS/patient/day and differed between PDP and OPDP (0.66 vs. 1.00). Daily utilization was greatest in patients receiving therapy associated with a pre-defined high risk of hypoglycemia [insulin: basal + bolus (2.16), premixed (1.65), basal (1.16), other insulin regimens (2.13), and sulfonylureas (0.74)] versus non-sulfonylurea non-insulin-based regimens (0.52). For non-insulin therapy, BGTS utilization was greater for patients on multiple non-insulin therapies versus monotherapy (0.74 vs. 0.53 TS/patient/day). In multivariate analyses, drivers for BGTS utilization included insulin use, previous BGTS use, and female gender. Previous diabetes therapy and duration of therapy were negatively correlated with BGTS utilization.

**Conclusions:**

BGTS utilization varies depending on the type of therapy used to treat T2D according to hypoglycemic risk. Decision making regarding BGTS needs to account for robust analyses of current utilization and its value in those settings, including in patients not receiving diabetes therapy and the prevalence of circumstances conducive to more intensive monitoring.

## Introduction

National and international clinical practice guidelines for the management of diabetes currently recommend individualized self-monitoring of blood glucose (SMBG) based on several factors, such as the patient’s type of diabetes and diabetes therapy regimen [1–7]. While patients with type 1 diabetes (T1D) require insulin, patients with type 2 diabetes (T2D) may manage their disease with diet, exercise, and a variety of treatments (e.g., non-insulin-based therapy, and insulin) [1–7]. SMBG allows for the detection of hyperglycemia and hypoglycemia to inform disease management [2, 3]. Compared with insulin, the risk of hypoglycemia associated with non-insulin therapies is lower and is generally limited to secretagogues [2, 3, 8, 9].

Given the cost of blood glucose test strips (BGTSs), the increasing global prevalence of T2D and the availability of new diabetes therapies with lower risk of hypoglycemia, there is considerable interest in ensuring the appropriate use of SMBG in patients with T2D. However, internationally, patient-reported BGTS utilization varies markedly [10]. Also, costs per test strip (TS) vary from $0.35 (Australia) to $3.11 (India) (adjusted to US dollar purchasing power, 2006) [10]. Recently, BGTS represented the third largest expenditure for the public drug formulary in Ontario, Canada [10, 11]. To economize SMBG, in 2009, the Canadian Agency for Drugs and Technologies in Health (CADTH) recommended limiting BGTS use to insulin-dependent and gestational diabetes while withdrawing it from most patients with T2D on non-insulin diabetes therapy or no therapy [12]. The CADTH recommendations were criticized by the Canadian Diabetes Association (CDA) for undervaluing the merits of self-management in non-insulin-treated patients and for not accounting for the increased risk of hypoglycemia associated with secretagogues [13]. Notably, Gomes et al. observed a 33 % increase in daily BGTS utilization for non-insulin therapies associated with hypoglycemia versus those not associated with hypoglycemia [14].

Despite this, important questions persist. There are limited data on differences in SMBG across specific regimens associated with differential hypoglycemic risk. Additionally, the prevalence and value of SMBG in patients with T2D not treated with a pharmacologic agent are not well understood. Further, there is a lack of information on the impact of alternative methods of analyzing BGTS utilization employed to date. The objective of this study was to build on the existing literature, to determine whether BGTS utilization in patients with T2D is associated with the type of diabetes therapy, classified according to hypoglycemic risk.

## Methods

Two claims databases were used to identify patients with T2D in Canada from January 1, 2006, to December 31, 2012. These two databases capture public (Ontario Public Drug Programs [OPDP]) and private (private drug plans [PDP]) market claims for BGTS. The OPDP covers approximately 2.5 million claimants in Ontario and 115 million prescriptions annually. The majority (68 %) of claimants in the OPDP are aged ≥65 years, with ~32 % on social assistance, disability, catastrophic illness, or other benefits [15]. The OPDP database has a 100 % capture rate. The PDP covers 10 million Canadians across Ontario (34 %), Quebec (28 %), Western Canada (29 %), and Atlantic Canada (9 %), and 100 million prescriptions annually. The PDP database has a 70 % capture rate.

### Study design

This study built on earlier methodology [12, 15] to elucidate the impact of study design and sampling on BGTS utilization. Previous studies assessed BGTS utilization in all patients with diabetes (T1D and T2D) who had at least one BGTS prescription claim, which does not account for differences in the two populations. Patients were assigned to yearly cohorts based on the most prominent diabetes therapy received during that period. Since patients may be on multiple therapies over the course of a year, a more specific sampling method may yield different results. Utilization was assessed according to diabetes therapy type; however, some studies classified all insulins in a single category [14, 16] and/or did not account for hypoglycemic risk associated with secretagogues [14]. Yet, unique regimens may be associated with different patterns of SMBG. Neither study accounted for non-BGTS users in its estimates of utilization.

**Fig. 1:**
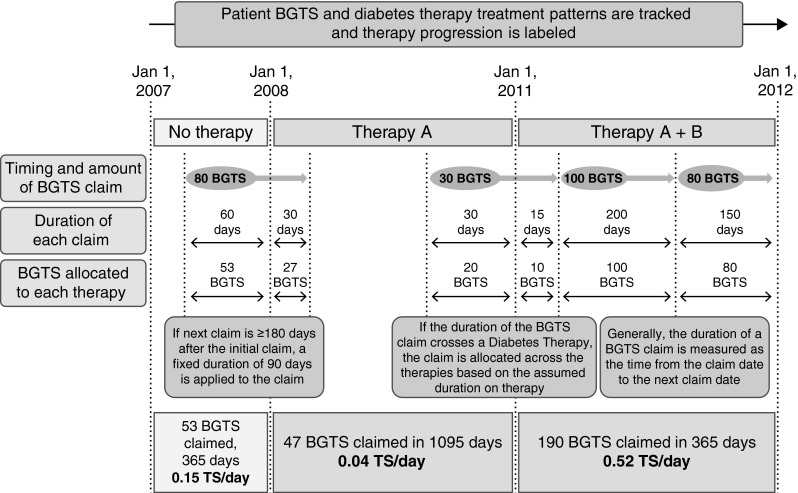
An illustration of the longitudinal approach to accounting for diabetes therapy and allocation of BGTS utilization *BGTS* blood glucose test strips, *TS* test strips

Given the above, once the results of the initial CADTH study [16] were reproduced, the following methodological changes were implemented and the independent effect of each explored: (1) addition of non-BGTS users; (2) limiting the population to patients with T2D according to the eligibility criteria listed below; (3) extending the study period through 2012; (4) using a longitudinal approach, to capture all diabetes therapies over the entire study timeframe, rather than attributing BGTS utilization to the dominant regimen (Fig. 1); and (5) combining points 1–4 in the overall analysis.

The longitudinal approach to measuring diabetes therapy allowed BGTS utilization to be attributed to unique regimens (i.e., multiple observations from a single claimant were possible; Fig. 1). Diabetes therapy and BGTS utilization were inferred from claims filed. Diabetes therapy claims were chronologically ordered per patient, with combination therapies determined by the overlapping dates for each agent. A treatment duration of 90 days from the therapy claim date was used as a default. Otherwise, a new diabetes therapy claim extended the treatment duration or signified a treatment addition. Where a delay of less than 60 days existed between the default duration expiry and a new claim extending the therapy, an adjustment was made to infer treatment continuity. A period of BGTS utilization (TS/day) was allocated to therapy using three rules based on the timing and duration of BGTS claims. If the time between BGTS claims was less than 180 days, the duration of a BGTS claim was determined as the time from one claim to the next. If there was a period of at least 180 days between claims, the duration of the initial claim was set at 90 days. Finally, if the duration of a BGTS claim spanned two diabetes therapies, the number of BGTS claimed was divided between regimens proportional to the number of BGTS claim days that overlapped with each therapy.

### Eligibility criteria

Patients were included in the analysis if they filed a claim for BGTS only (with no diabetes therapy), a non-insulin diabetes therapy, or any basal, rapid, or premixed insulin (human or analog). Those claiming either basal insulin only continuously, premixed monotherapy at any point in the analysis period, or a non-insulin diabetes therapy at any point in the study period, were classified as T2D. Non-insulin diabetes therapy included metformin (Met), secretagogues (sulfonylureas [SU] and postprandial glucose regulators [PPGR]), thiazolidinediones (TZD), dipeptidyl peptidase-4 inhibitors (DPP4), prandase, and glucagon-like peptide-1 receptor agonists (GLP-1). As determined through historical product use, patients on insulin-only regimens that were not classified as T2D (i.e., patients with T1D) were excluded. Patients who were new to the database in the 6 months prior to their first claim were also excluded from the analysis.

### Statistical analyses

Mean BGTS utilization was reported as per patient per day (TS/patient/day) by type of diabetes therapy. Given the extent of data capture, the sample means reported are nearly exact point estimates of the population mean. Diabetes therapies were classified according to risk of hypoglycemia. Non-insulin diabetes therapies, basal insulin only, premixed insulin only, and prandial insulin regimens were all considered to be associated with differential BGTS utilization due to the heterogeneous risk for hypoglycemia. At the cohort level, per patient daily BGTS utilization was compared across these categories of therapy.

Multivariate analyses were conducted to identify additional determinants of BGTS utilization; the variables considered are listed in Table 1. The cohort characteristics of ‘*Public*’ and ‘*Private*’ were too heterogeneous to combine the datasets (e.g., age, socioeconomic status, and drug coverage). Experience with previous diabetes therapy (‘*Diabetes Treatment Experience*’) or BGTS (‘*BGTS Experience*’) was determined by checking for previous diabetes therapy or BGTS claims in the 6 months prior to the first claim during the study. The ‘*Experience*’ of patients who did not have a previous claim was classified as ‘*Naïve*’ versus ‘*Experienced*.’ The ‘*Event* (*over time*)’ variable was included to explore the effect of key research findings on BGTS utilization [14, 16–20]. An approximate cutoff date of July 2009 was used to account for the potential effect of both the ACCORD study [17], which found that intensive glycated hemoglobin control increased mortality and did not lower cardiovascular risk, and studies investigating the utilization and value of SMBG [12, 14, 16, 18–20].

**Table 1:** Cohort variables considered for multivariate analysis

Cohort variables	Description
Diabetes drug	Met, SU, PPGR, TZD, DPP4, prandase, GLP-1^a^, basal, rapid, premixed
Diabetes class	Insulin therapy, non-insulin therapy, both, none
Gender	Male, female, unknown
Age group, years	<20, 20–39, 40–59, 60–79, 80+
Payer	Public, private
Province	PDP cohort only
Diabetes treatment experience	Naïve, experienced
BGTS experience	Naïve, experienced
Event (over time)	Research findings on BGTS (i.e., ACCORD, CADTH recommendations) (Before July 2009, after July 2009)
Duration on line of therapy	Continuous (no. of days)
Time on therapy	Continuous (no. of days)
Time on any therapy	Continuous (no. of days)
Related complications	All, some, none (anti-infective, hypertension, dyslipidemia, ophthalmic)
Interaction	Between therapies

*BGTS* blood glucose test strips, *CADTH* Canadian Agency for Drugs and Technologies in Health, *DPP-4* dipeptidyl peptidase-4, *GLP-1* glucagon-like peptide-1 agonist, *Met* metformin, *PDP* private drug plans claims database, *PPGR* postprandial plasma glucose regulator, *SU* sulfonylureas, *TZD* thiazolidinedione

^a^GLP-1 included in PDP only

After eliminating redundant/highly correlated variables (e.g., ‘*Time on any Therapy*’ removed as it was correlated with ‘*Duration of Therapy*’) and exclusion of others with limited rationale (e.g., ‘*Related Complications*’), the following were included in the final multivariate analysis: diabetes product [*Met, SU, PPGR, TZD, DPP4, prandase, GLP*-*1* (for PDP dataset only)*, Basal, Rapid, Premixed*], diabetes therapy class (*insulin therapy, non*-*insulin therapy, both, none*), *Gender, Age Group, Province* (for PDP dataset only), *Diabetes Treatment Experience*, *Duration of Therapy, BGTS Experience*, and *Event* (*over time*).

The final multivariate analyses were completed on a random sample of 0.5 % of the full cohort to evaluate the significance of determinant effects in a clinically meaningful sample size. To adjust for the correlation of utilization within patients, a generalized linear mixed model was used [21]. All statistical analyses were conducted in SAS/STAT version 9.3 (Cary, NC). Adjusted means are presented to account for different patient dependencies.

## Results

Compared with earlier studies from a similar sample [12, 14, 16], the method adopted in the current study resulted in lower estimates of overall mean daily BGTS utilization (Table 2). In order to estimate the effect of each revised step to the methods, the results from the earlier study were approximately replicated across the therapeutic categories [16]. The original study reported a total mean BGTS utilization of 1.47 TS/patient/day across 472,578 patients. Replication of the cohort resulted in a total mean BGTS utilization of 1.45 TS/patient/day across 481,913 patients. The inclusion of BGTS non-users or using the longitudinal approach each independently resulted in markedly lower estimates of BGTS utilization (1.01 and 1.02 TS/patient/day, respectively). The effects of other steps were less pronounced. For insulin-alone claimants, limiting the cohort to T2D lowered average utilization from 2.94 to 2.81 TS/patient/day. Utilization estimates for other treatment categories were unaffected. Extending the cohort window from 2006 to 2012 resulted in a greater estimate of utilization among insulin-alone claimants from 2.94 to 3.10 TS/patient/day. Total and other therapy-specific mean estimates of BGTS utilization closely approximated the original 2006 estimates. The cumulative effect of the revised methods was BGTS utilization estimates that were less than those produced from any individual, independent revision. The total mean BGTS utilization from 2006 through 2012 measured using the revised method was 0.84 TS/patient/day.

**Table 2:** Mean daily BGTS utilization applying different methodological considerations

Sample description		Sample (*n*)	BGTS utilization (% cohort)				
			No therapy	Non-insulin alone	Insulin + non-insulin (both)	Insulin alone	Total
Base	BGTS users, 2006^a^	OPDP, PDP, 2006 only (472,578)	0.85 (18)	1.19 (57)	2.15 (11)	2.99 (14)	1.47 (100)
	BGTS users, 2006	OPDP, PDP, 2006 only (481,913)	0.82 (19)	1.17 (56)	2.11 (11)	2.94 (14)	1.45 (100)
Change applied to base	1. Add in non-BGTS users	OPDP, PDP, 2006 only (693,178)	0.82 (13)	0.69 (66)	1.77 (9)	2.35 (12)	1.01 (100)
	2. Limit to T2D	OPDP, PDP, 2006 only (476,386)	0.82 (19)	1.17 (57)	2.10 (11)	2.81 (13)	1.42 (100)
	3. Extend study period	OPDP, PDP, 2006–2012 (3,530,286)	0.81 (18)	1.17 (55)	2.12 (13)	3.10 (14)	1.50 (100)
	4. Longitudinal approach	OPDP, PDP, 2006 only (1,037,088)	0.82 (21)	0.76 (55)	1.75 (9)	2.46 (15)	1.02 (100)
	5. 1–4 combined	OPDP, PDP, 2006–2012 (5,759,591)	0.78 (24)	0.61 (52)	1.59 (10)	1.81 (13)	0.84 (100)

*BGTS* blood glucose test strips; *CADTH* Canadian Agency for Drugs and Technologies in Health, *OPDP* Ontario Public Drug Programs claims database, *PDP* private drug plans claims database, *T2D* type 2 diabetes

^a^Values in row one are taken from the CADTH report [18] and are provided for comparison only

### Cohort characteristics

The T2D sample was comprised of 1,949,129 claimants, including 63.7 % (*n* = 1,241,086) from the PDP cohort (Table 3). The OPDP cohort included similar percentages of males and females, and 44.7 % of patients had age missing from their records. The PDP cohort consisted of a greater percentage of males (53.0 %), and the majority of patients were in the 40–59 (43.6 %) and 60–79 (42.3 %) age groups. The distribution of diabetes therapy was similar across both cohorts with the majority of patients clinically managed using non-insulin diabetes therapy alone (OPDP, 55.1 %; PDP, 53.7 %). The percentage of BGTS users not on a diabetes therapy (27.4 %) far outnumbered those on an insulin-only regimen (7.3 %) and remained greater even when those using non-insulin diabetes therapy in combination with insulin were factored in (18.4 %). A greater percentage of patients in the OPDP cohort were on an insulin and non-insulin diabetes therapy regimen (OPDP, 15.4 %; PDP, 8.6 %). Across both cohorts, the majority of patients entered the study previously using neither diabetes therapy (*Diabetes Treatment Experience* ‘*Naïve*’: OPDP, 64.3 %; PDP 81.4 %) nor BGTS (*BGTS Experience* ‘*Naïve*’: OPDP, 75.3 %; PDP, 90.9 %).

**Table 3:** Patient cohort characteristics at study inclusion

Characteristic, %	Unique claimants		
	OPDP (*n* = 708,043)	PDP (*n* = 1,241,086)	Total (*n* = 1,949,129)
Gender			
Female Male Unknown	49.7 49.3 1.0	46.3 53.0 0.6	47.5 51.7 0.8
Age group, years			
<20 20–39 40–59 60–79 80+ Unknown	0.1 1.1 6.3 15.1 32.7 44.7	1.0 10.7 43.6 42.3 2.3 0.0	0.6 7.2 30.0 32.5 13.4 16.2
Diabetes therapy			
Non-insulin alone Insulin + non-insulin (both) Insulin alone None	55.1 15.4 6.8 22.6	53.7 8.6 7.6 30.2	54.2 11.1 7.3 27.4
Diabetes treatment experience			
Naïve Experienced	64.3 35.7	81.4 18.6	75.2 24.8
BGTS experience			
Naïve Experienced	75.3 24.7	90.9 9.1	85.2 14.8

*BGTS* blood glucose test strips, *OPDP* Ontario Public Drug Programs claims database, *PDP* private drug plans claims database

### Bivariate analysis

Each claimant may have contributed more than one observation depending on the number of unique treatment regimen courses used over follow-up. Whereas Table 3 reports the distribution of sample characteristics by claimant, bivariate and multivariate analyses accounted for a total of 5,759,591 observations by unique treatment regimen course used over follow-up.

Overall, BGTS utilization was greatest in claimants receiving therapy associated with a pre-defined high risk of hypoglycemia (e.g., SU, basal + bolus insulin regimens; Table 4). For claimants on any non-insulin therapy, BGTS utilization was greater for those on multiple non-insulin therapies, compared with others on one non-insulin therapy (0.74 and 0.53 TS/patient/day, respectively). Use of SU was associated with greater BGTS use, compared with non-insulin-non-SU therapies (0.74 and 0.52 TS/patient/day, respectively). For claimants on any insulin therapy, BGTS utilization was greater for those on insulin-only therapy, compared with others on both insulin and non-insulin therapies (1.81 and 1.59 TS/patient/day, respectively). BGTS utilization was greatest for claimants on basal + bolus regimens, followed by those on premixed insulins and others on basal-only regimens.

**Table 4:** BGTS utilization across therapy classes for T2D among claims beneficiaries in the OPDP and PDP^a^

Therapy description			Mean BGTS utilization, TS/patient/day [%]		
			OPDP (*n* = 2,486,394)	PDP (*n* = 3,273,197)	Total (*n* = 5,759,591)
No therapy^b^			0.82 [21.3]	0.75 [26.5]	0.78 [24.3]
Non-insulin-based regimens^c^	Non-SU	Monotherapy	0.67 [24.0]	0.36 [28.6]	0.51 [26.6]
		+non-SU, non-insulin	0.84 [3.5]	0.45 [5.6]	0.58 [4.7]
		Total	0.68 [27.5]	0.37 [34.2]	0.52 [31.3]
	SU	Monotherapy	0.74 [7.3]	0.35 [4.7]	0.61 [5.8]
		+non-insulin	0.96 [18.3]	0.51 [13.0]	0.78 [15.3]
		Total	0.90 [25.6]	0.48 [17.7]	0.74 [21.1]
Insulin-based regimens	Basal insulin	Monotherapy	1.52 [3.3]	0.84 [5.0]	1.11 [4.3]
		+non-insulin	1.50 [5.1]	0.89 [4.3]	1.21 [4.6]
		Total	1.51 [8.4]	0.87 [9.3]	1.16 [8.9]
	Basal + bolus insulin	Monotherapy	2.68 [3.3]	1.98 [4.6]	2.24 [4.1]
		+non-insulin	2.44 [2.3]	1.64 [2.4]	1.99 [2.4]
		Total	2.60 [5.6]	1.87 [7.0]	2.16 [6.4]
	Premixed insulin	Monotherapy	1.83 [3.6]	0.94 [1.3]	1.64 [2.3]
		+non-insulin	1.86 [3.0]	1.10 [1.0]	1.68 [1.9]
		Total	1.84 [6.7]	1.01 [2.3]	1.65 [4.2]
	Other insulin^d^	Monotherapy	2.44 [2.8]	2.00 [2.0]	2.24 [2.4]
		+non-insulin	2.14 [2.1]	1.50 [1.0]	1.91 [1.5]
		Total	2.33 [4.9]	1.86 [3.0]	2.13 [3.8]

*BGTS* blood glucose test strips, *OPDP* Ontario Public Drug Programs claims database, *PDP* private drug plans claims database, *SU* sulfonylureas, *TS* test strips, *T2D* type 2 diabetes

^a^Observations represent unique treatment regimens (i.e., multiple observations per claimant are possible)

^b^Excludes patients with prediabetes or T2D not using BGTS

^c^‘Non-insulin’ includes oral anti-diabetic therapies and glucagon-like peptide-1 receptor agonists

^d^‘Other insulin’ includes combinations of insulin-based regimens

Generally, the differences observed were consistent for both databases. Utilization of BGTS tended to be greater in the OPDP database, compared with the PDP database (overall: 1.00 TS/patient/day and 0.66 TS/patient/day, respectively), irrespective of therapy type. For claimants not on any diabetes therapy, BGTS utilization averaged 0.78 (OPDP, 0.82; PDP, 0.75) TS/patient/day.

### Multivariable analysis

Across a random 0.5 % sampling of each cohort, multivariable-adjusted differences between the diabetes therapy classifications (insulin alone, insulin + non-insulin (both), non-insulin, and none) were statistically significant (*P* < 0.0001 for both cohorts). The significance of differences between insulin alone and insulin + non-insulin (both) treatment regimens was inconsistent across the two cohorts. Differences between insulin-based regimens (insulin alone or insulin + non-insulin [both]) and either non-insulin or no therapy were generally significant (*P* < 0.0005). Using this sampling method, there were insufficient data to determine a significant statistical difference between insulin alone and no therapy in the PDP cohort (*P* = 0.08). Similarly, there were insufficient data to determine significant statistical differences between specific non-insulin therapies in the analysis conducted.

Across the OPDP and PDP multivariable predictive models developed, a number of variables were consistently and significantly associated with BGTS utilization. Insulin use (*Basal*, *Rapid*, *Premixed*), *BGTS Experience*, and *Gender* (female) had independent, positive effects on BGTS utilization. In contrast, *Diabetes Treatment Experience* and *Duration of Therapy* negatively impacted BGTS utilization. Other variables including specific non-insulin therapies, *Age* and *Event* (*over time*), had inconsistent effects across the two cohorts.

## Discussion

The utilization of BGTS in patients with T2D receiving diabetes therapy associated with hypoglycemia exceeds that of patients receiving other diabetes therapies. Consistent with earlier findings [14] and guideline recommendations [1], at the population level BGTS utilization was greater for secretagogue-containing non-insulin regimens, compared with other non-insulin regimens. The utilization of BGTS was greater for more complex insulin-based regimens requiring multiple daily injections, with basal + bolus regimens achieving the highest utilization. Additionally, the study demonstrated substantial SMBG among patients not on a diabetes therapy. As previously noted [12, 14, 16, 22], the frequency of use generally increased with the intensity of the treatment regimen, from non-insulin to insulin regimens. In multivariate analyses, drivers for BGTS utilization in both public and private claims databases included insulin use, previous BGTS use, and female gender. Previous diabetes therapy and duration of therapy were negatively correlated with BGTS utilization.

For therapies associated with hypoglycemia, the risk of a hypoglycemic event exists with the administration of each dose. Perhaps for this reason, Canadian guidelines recommend SMBG at least as frequently as insulin is administered [1]. Bolus insulin therapy is administered several times a day with each meal, premixed insulin is administered twice daily, and basal insulin regimens are typically administered once or twice daily [23]. The current study showed that patients with T2D managed with basal–bolus insulin regimens use up to a mean of 2.16 TS/patient/day, followed by premixed insulin (1.65 TS/patient/day), and basal insulin (1.16 TS/patient/day). The difference relative to guidelines was most pronounced among patients on private health plans. The findings indicate that BGTS utilization for bolus and premixed regimens is lower than recommended in these patients.

The utilization of BGTS in patients on a basal insulin regimen exceeding the minimum recommendation may be explained by the utilization of specific products and their properties. Over the timeframe of this study, the proportion of basal insulin prescriptions for neutral protamine Hagedorn (NPH) insulin was substantial, ranging from 71 % in 2007 to 40 % in 2012 [24]. Twice-daily injection of NPH insulin is commonly required to provide 24-hour basal coverage due to its pharmacokinetic properties [25]. Taken together with the guideline recommendations, the dosing frequency of NPH insulin may contribute to BGTS utilization among patients treated with basal insulin alone exceeding the default minimum of one TS/patient/day. Further, NPH insulin is associated with an increased risk of hypoglycemia relative to other basal insulin regimens [26, 27], independent of its greater dosing frequency [28]. The prevalent use of NPH insulin in the study setting may have contributed to the mean BGTS utilization observed.

Conversely, the Canadian guidelines do not specify a frequency of testing for patients with T2D managed without insulin, but instead suggest that the testing should be individualized [1]. In the current study, those managed without insulin used 0.61 TS/patient/day. The data from this study may reflect the individualized nature of BGTS utilization according to the type of non-insulin therapy, with patients on SU using more BGTS.

The utility of SMBG in T2D treated with non-insulin antidiabetic therapy is tenuous [1]. Despite the debate, systematic reviews of the literature demonstrate a finite benefit for glycemic control associated with SMBG [29–32]. Health technology assessments from different regions report conflicting conclusions regarding the cost-effectiveness of SMBG for patients with T2D treated with non-insulin therapies using conventional willingness-to-pay thresholds [33–36]. The implications of SMBG on glycemic control and hypoglycemic events are controversial or not well studied [16, 29, 37].

Differences in BGTS utilization among T2D were investigated employing alternative methods relative to existing studies. The methodology used in this study builds on approaches used previously to assess BGTS utilization [14, 16]. Compared with earlier studies, unique patient treatment regimens were analyzed to estimate utilization from nearly 6 million observations. In this study, BGTS utilization over 7 years was investigated. Patients with diabetes who did not use BGTS were also analyzed, as were patients utilizing BGTS without concomitant use of diabetes therapy, to allow for a more accurate estimation of average BGTS utilization across the diabetes population. When non-BGTS users were included, estimates of the average utilization were lower than those predicted by Gomes et al. (1.22 TS/patient/day) and CADTH (1.38–1.56 TS/patient/day) [14, 16]. Including these patients accounted for the increasing proportion of patients on insulin who included SMBG as part of their diabetes management. Not accounting for this biases utilization estimates among non-insulin therapy users to greater values. The current analysis accounted for more specific use of SMBG in the clinical management of T2D, which may be important when considering the policy implications. Additionally, the population was limited to patients with T2D. This approach allowed several novel explorations of determinants of BGTS utilization such as *BGTS Experience*, *Diabetes Treatment Experience,* and *Duration of Therapy*.

Further research is required to understand the effect of other variables on BGTS utilization. Previous experience with BGTS generally increased utilization, which may be due to familiarity and patient satisfaction with SMBG. Alternatively, greater utilization among patients previously using BGTS may represent bias toward those adherent to SMBG. The observation that BGTS utilization decreased with duration of therapy was consistent with the general finding of poor SMBG adherence [38]. Experience with self-management of the disease may also contribute to a decreased dependence on SMBG. The effect of these changes to SMBG on patient outcomes is largely unknown.

A few limitations of the study were considered. First, BGTS utilization and diabetes therapy data were limited to claims for these benefits. As such, they may neither account for wastage nor reflect exact usage [39]. Second, BGTS utilization for patients funding treatment themselves could not be included in the analyses. Likewise, unfiled PDP claims were not captured. Third, both databases contain distinct population samples with the potential for duplicate patients in both datasets. From a payer perspective, the claims analysis approach accounted for the healthcare resource utilization associated with BGTS. Further work is required to understand BGTS utilization from a societal perspective. Finally, in the current study, private and public plans were not directly compared. Differences in the demographics and level of coverage between the two insurance plans could affect utilization. For instance, the private database contained younger working-age patients who typically have higher co-payments than patients on public drug plans and therefore may be less likely to refill prescriptions. A recent study evaluating BGTS utilization has indicated that patients use more TS if they are more easily accessible [40]. In this multivariate analysis, drivers for BGTS utilization were similar for the public and private cohorts; however, magnitudes of utilization differed.

Overall, the results of this study show that BGTS utilization varies depending on the type of therapy used to treat T2D. Decision making regarding BGTS needs to take into account robust analyses of current utilization, including the roles of SMBG in prediabetes or patients on no diabetes therapy, as well as an understanding of the prevalence of individually unique circumstances conducive to more intensive monitoring. Only then can BGTS use, and subsequently costs, be contained without compromising patient outcomes.
